# Supplementary material for: A New Perspective: Revealing the Algicidal Properties of Bacillus subtilis to Alexandrium pacificum from Bacterial Communities and Toxins
Source: Mar Drugs. 2022 Sep 30;20(10):624. doi: 10.3390/md20100624 (PMC9605167; doi:10.3390/md20100624)
Supplement: Supplementary file 1 [file marinedrugs-20-00624-s001.zip › marinedrugs-1913838-supplementary.pdf]

Supplementary material S1

- In order to state that there were statistically significant differences in the increase of GTX2/3 and GTX5, and the decrease of GTX1/4.

Table S1. Changes in the content of GTX1/4, GTX2/3, and GTX5 with treatments at 0 h, 3 h, and 12 h. CK—control algae untreated; A—algae treated with 0.5% (v/v) B. subtilis S3 bacterial culture; B—algae treated with 0.5% (v/v) bacterial resuspension; C—algae treated with 0.5% (v/v) sterile supernatant; D—algae treated with 0.5% (v/v) LB liquid medium.

PSTs		GTX1	GTX4	GTX2	GTX3	GTX5
CK	**0 h**	15.15±2.11	8.58±1.24	2.93±0.84	1.52±0.25	4.55±0.49
	**3 h**	14.59±1.08	8.70±5.45	3.82±2.72	2.20±1.74	5.74±4.00
	**12 h**	13.93±1.50	9.27±0.97	3.09±1.78	1.50±0.91	5.02±3.70
A	**0 h**	16.99±3.62	10.26±1.79	1.24±0.04	0.66±0.12	4.73±0.21
	**3 h**	15.62±0.03	9.80±1.14	3.97±0.11	2.15±0.13	6.80±0.52
	**12 h**	9.53±1.64**	6.38±0.73**	8.58±0.15**	4.61±0.28**	12.11±0.17**
B	**0 h**	22.21±0.00	12.81±0.47	2.38±0.07	1.56±0.23	5.50±0.55
	**3 h**	19.94±3.25	10.95±1.00	3.91±0.38	2.40±0.12	5.56±1.56
	**12 h**	18.64±0.67	14.00±1.65	3.89±1.05	2.41±1.04	4.88±0.64
C	**0 h**	19.37±1.78	9.90±0.67	1.39±0.12	0.77±0.13	4.08±0.11
	**3 h**	16.21±2.62	9.68±1.04	3.19±1.07*	1.90±0.79*	6.18±0.19
	**12 h**	12.19±1.41*	5.77±1.09*	7.63±0.85**	4.04±0.38**	12.95±0.14**
D	**0 h**	21.76±0.93	10.96±1.33	2.54±0.41	1.33±0.51	5.36±0.43
	**3 h**	20.15±1.56	12.90±0.77	2.63±0.35	1.63±0.32	6.64±0.22
	**12 h**	21.04±2.90	14.29±0.88	4.63±0.60**	2.76±0.25*	7.33±0.07

Note: Data represent the mean + SD of triplicate measurements (n = 3). One-way ANOVA is used to analyze the difference between the control group and treatment groups, "*" indicates a significant difference ($p < 0.05$), and "**" indicates an extremely significant difference ($p < 0.01$).

Supplementary material S2

- provided HPLC chromatograms of the PSTs analysis standards and one of the samples (The intracellular PSTs of group A-3: *A. pacificum* treated with 0.5% of *B. subtilis* S3 bacterial culture for 3 h) as follows:

- PSTs standards analysis:

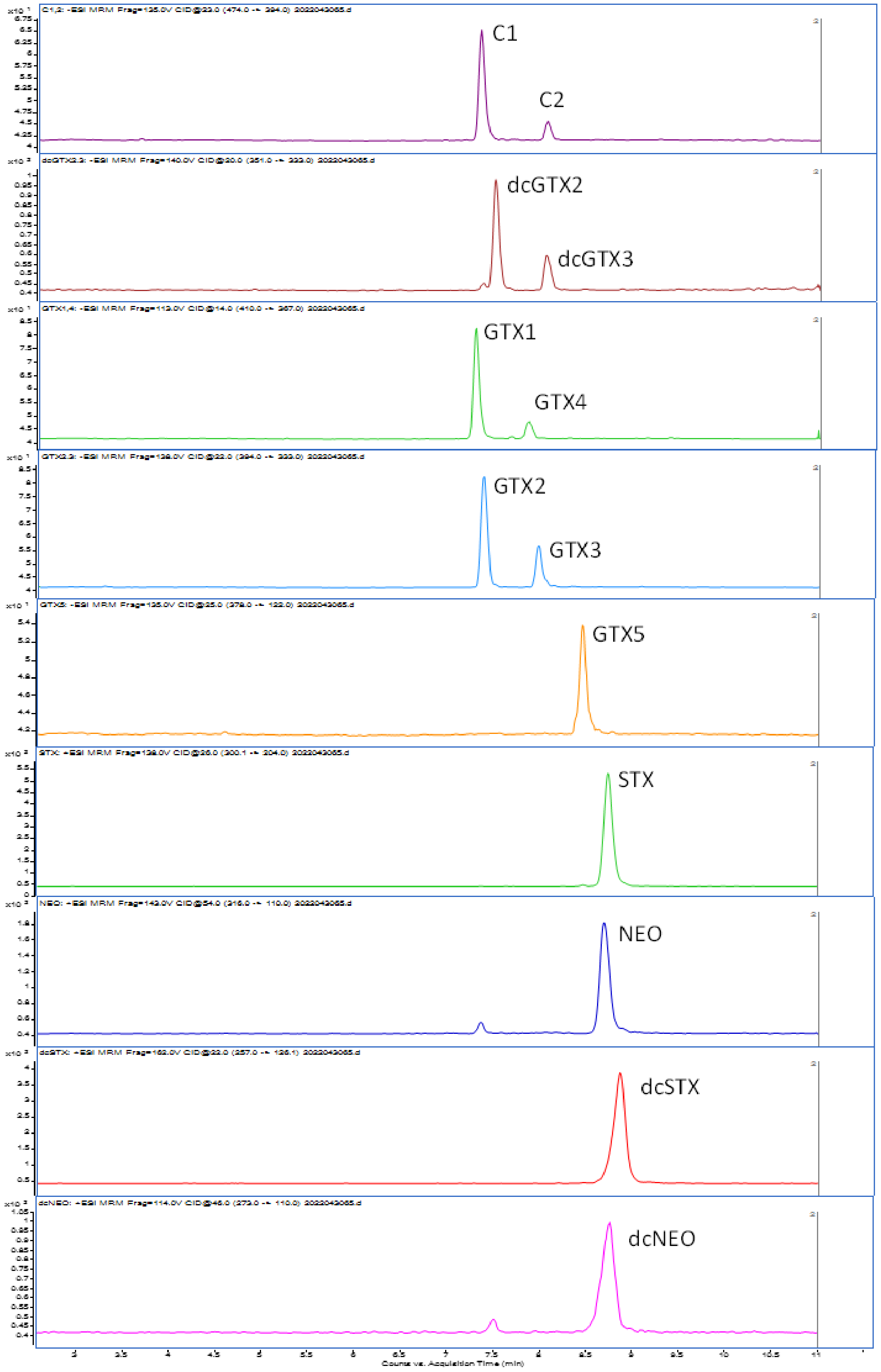

- The intracellular PSTs of group A-3 analysis:

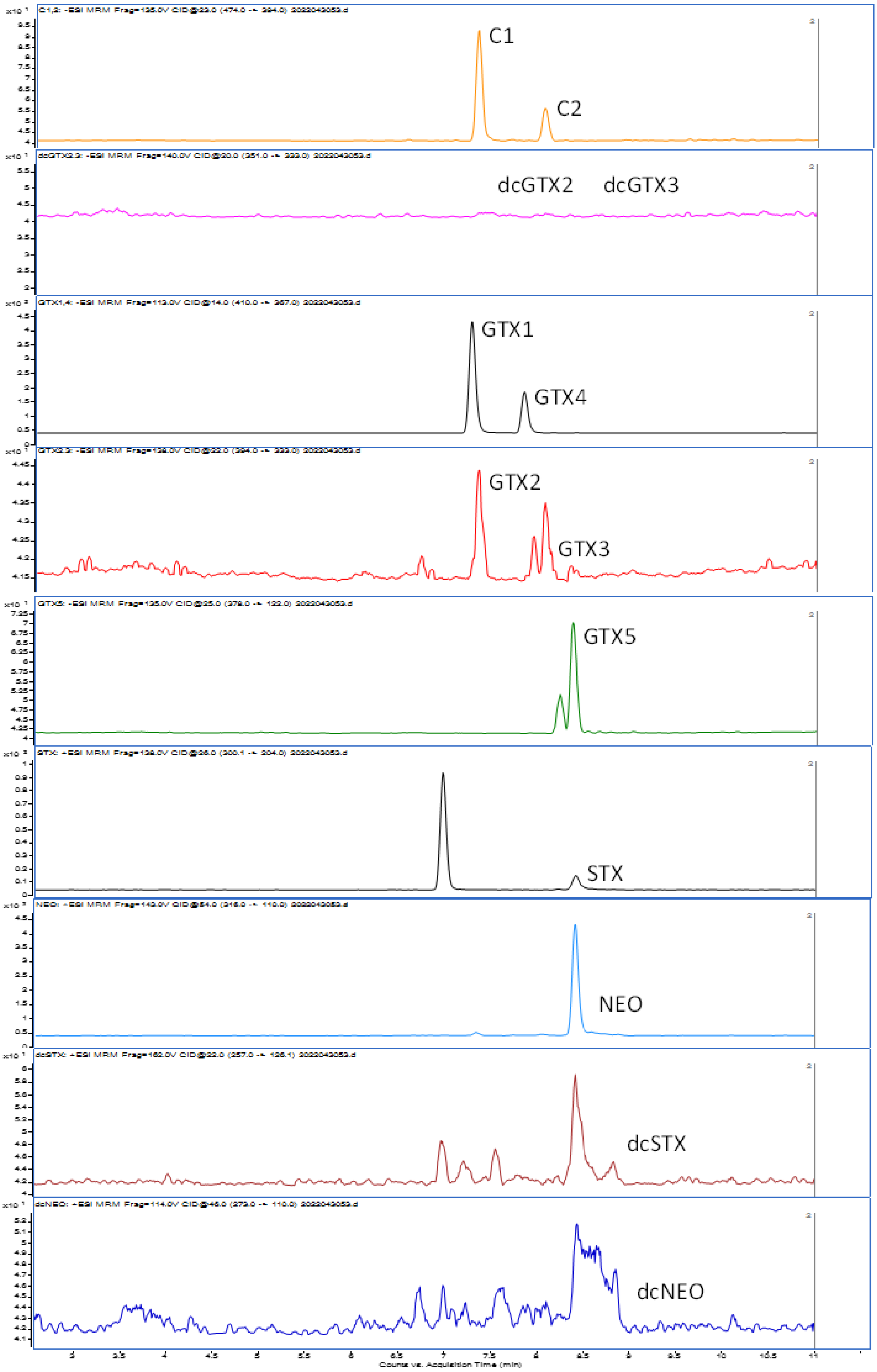

Figure S1. HPLC chromatograms of the PSTs standards and one of the samples (the intracellular PSTs of group A-3: *A. pacificum* treated with 0.5% of *B. subtilis* S3 bacterial culture for 3 h).
